# Analysis of equity and social inclusiveness of national urban development policies and strategies through the lenses of health and nutrition

**DOI:** 10.1186/s12939-021-01439-w

**Published:** 2021-04-16

**Authors:** Obinna Onwujekwe, Chinyere Ojiugo Mbachu, Chukwuedozie Ajaero, Benjamin Uzochukwu, Prince Agwu, Juliana Onuh, Charles Tochukwu Orjiakor, Aloysius Odii, Tolib Mirzoev

**Affiliations:** 1 Faculty of Medicine, College of Medicine, University of Nigeria, Nsukka, Nigeria; 2 Health Policy Research Group, University of Nigeria, Nsukka, Nigeria; 3 Faculty of Social Sciences, University of Nigeria, Nsukka, Nigeria; 4 Nuffield Center for International Health, University of Leeds, Leeds, UK

**Keywords:** Equity, Health, Inclusion, Nutrition, Urban development

## Abstract

**Introduction:**

Rapid urbanization increases competition for scarce urban resources and underlines the need for policies that promote equitable access to resources. This study examined equity and social inclusion of urban development policies in Nigeria through the lenses of access to health and food/nutrition resources.

**Method:**

Desk review of 22 policy documents, strategies, and plans within the ambit of urban development was done. Documents were sourced from organizational websites and offices. Data were extracted by six independent reviewers using a uniform template designed to capture considerations of access to healthcare and food/nutrition resources within urban development policies/plans/strategies in Nigeria. Emerging themes on equity and social inclusion in access to health and food/nutirition resources were identified and analysed.

**Results:**

Access to health and food/nutrition resources were explicit in eight (8) and twelve (12) policies/plans, respectively. Themes that reflect potential policy contributions to social inclusion and equitable access to health resources were: Provision of functional and improved health infrastructure; Primary Health Care strengthening for quality health service delivery; Provision of safety nets and social health insurance; Community participation and integration; and Public education and enlightenment. With respect to nutrition resources, emergent themes were: Provision of accessible and affordable land to farmers; Upscaling local food production, diversification and processing; Provision of safety nets; Private-sector participation; and Special considerations for vulnerable groups.

**Conclusion:**

There is sub-optimal consideration of access to health and nutrition resources in urban development policies in Nigeria. Equity and social inclusivity in access to health and nutrition resources should be underscored in future policies.

**Supplementary Information:**

The online version contains supplementary material available at 10.1186/s12939-021-01439-w.

## Introduction

As cities in Nigeria continue to grow, the demand and pressure on social and public services are expected to rise, and ensuring equitable access to priority services such as healthcare, food and nutrition services pose challenges to social inclusion. Nigeria is not only Africa’s most populous country, but also has more number of large cities and the highest urban population in sub-Saharan Africa [1]. In 2010, 62.5 m people were added to Nigerian cities, and it is forecasted that an additional 226 m more would be added by 2050 [2]. On the global hunger index rating, Nigeria ranks 93 of 117 countries, with a score of 27.9 [3].

A recent national survey revealed that socioeconomic inequalities are huge across urban settings of Nigeria suggesting that many urban dwellers do not have access to key resources and services [4, 5]. Disparities exist in the nutritional status of the urban-poor, urban-middle, and urban-rich. More cases of malnutrition and thinness have been noted among the urban-poor than their counterparts, the urban-middle and urban-rich [6]. Vulnerable groups are oftentimes most affected by poverty, rising unemployment, poor living conditions, and low development indicators [7]. Very often, they lack access to health care services just because they are unable to afford financial resources to pay for the care. This results in millions of people who are denied nutrition and life-saving health services. Recent estimates suggest that over 100 million Nigerians are poor [5].

The most vulnerable group that are prone to social exclusion in Nigeria include children, pregnant women, those with disabilities, elderly persons, displaced, unemployed, retirees, and the sick [8]. Inequality reduces opportunities and contributes to high rate of maternal and child mortality especially among the urban poor, underserved, and vulnerable groups [9]. The levels of social inclusion do vary from region to region and state to state thus tackling its concerns should be context-specific. Therefore, policies that consider these peculiarities and incorporate social inclusion are inevitable in the sustainable cities’ agenda.

Nigeria has developed policies and plans that provide strategic directions for more sustainable urban development and planning. A few of these policies such as the National Urban Development Policy (2012), National Social Protection Policy (2016), and the very recent Economic Sustainability Plan (2020) additionally seek to achieve social inclusion and equitable access to resources. The National Urban Development Policy was developed in 2012, with the goal to improve standard of healthy living and wellbeing for all Nigerians by promoting a system of well-managed urban settlements. In 2019, the Federal Government of Nigeria commenced plans to review and revise the policy and provide a governance framework for ensuring more inclusive and sustainable urban development. In order to ensure that a revised urban development policy makes provisions for social inclusion and equitable access to public resources for urban dwellers, a critical review of existing policies and plans is imperative. This will highlight any deficiencies, and enable a more focused and effective policy revision.

The effectiveness of urban development policies and strategies can be measured through the lens of social inclusion, which refers to the extent to which poor and vulnerable groups can easily access and benefit from urban resources. Social inclusion is a multifaceted construct, which forms the framework for overall well-being and enables community members to benefit as well as contribute meaningfully to community development [10]. Inclusion involves complex interrelationships between environmental factors and individual characteristics, which create opportunities to access public goods and services, as well as to experience and perform valued and expected social roles in ones community [11]. Furthermore, social inclusion has been conceptualized as the ability of individuals to participate in and benefit from society through three interrelated domains of markets (labor, land, housing, credit), spaces (political, cultural, physical, social), and services such as electricity, education, water and health [12]. On the flipside, social exclusion increases poverty, by reducing access to vital services such as nutrition, health, education, and livelihood opportunities. Hence, sustainable and inclusive urban growth must ensure equitable access to quality health care services and nutrition resources. And urban development plans should be aligned to guarantee access for all citizens, especially the most vulnerable [13, 14].

It is important to examine the extent that national policies, strategies and plans exist and how they address the issues of equity and social inclusion (i.e. promote or constrain equitable access to healthcare and food/nutrition). Evidence on social inclusion in urban development is critical for shaping evidence-informed policies and resource allocation that can make cities in rapidly urbanizing low and middle income countries (LMICs) socially inclusive, especially with regards to critical resources of health and food/nutrition. However, social inclusion in urban development is underresearched. Moreso, there is limited evidence from policy reviews to underscore the need to mainstream equity and social inclusion within public sector policies in Nigeria.

This study fills gaps in knowledge that are important to inform decisions that promote equity and social inclusion in urban development in LMICs such as Nigeria that are undergoing rapid urbanization. This paper examines the degree to which urban development policies, plans and strategies in Nigeria address equitable access and social inclusion in health and food/nutrition resources in the country. It highlights important policy gaps that need to be addressed in order to pave way for equitable access to urban resources for urban dwellers in the current mileu of rapid urbanization in Nigeria. These findings could inform policy revisions that are better aligned to the 2030 agenda for sustainable and inclusive cities.

## Methods

This study was undertaken using desk review of national and subnational policies, strategies, and plans for sustainable urban development in Nigeria. The document review included only policy documents that were published within 1999–2020 and written in English language. The retrospective review was limited to 20 years in order to reduce the chance of including obsolete policies and plans. However, the Urban and Regional Planning Act which was published in 1992 was included in the review because there has not been a review of the Act.

Although the scope of these documents covered urban areas, most of them were not developed explicitly for urban areas.

### Document search and retrieval

The search for electronic and paper-only documents was performed from March to May 2020. Both electronic and paper-only copies of documents were examined. Electronic searches were performed on the websites of government agencies including Ministries of Housing, Environment, Lands and Urban Planning, and Office of SDGs in Nigeria; as well as websites of international organizations including UN-Habitat, UNDP, World Bank, UNFPA, AfDB and UNICEF. The electronic search was performed using various combinations of these keywords

“urban”, “city”, “metropolitan”, “development”, “sustainable”, “sustainability”, “policy”, “plan”, “strategy”, “program”, “road map”, “guideline”.

Individual visits were made to key government agencies to source for policy documents and plans that were not available online.

The executive summary of each document was read to elicit if one of its goals is to contribute to urban development and planning in Nigeria. A total of 22 documents, comprising 5 policies and 17 plans/strategies/roadmaps, were identified. A descriptive summary of the geographic scope, policy goals, and sectors addressed in the policy documents is attached as supplemental file 1.

### Data extraction and analysis

Data were extracted verbatim from the source documents by six (6) independent reviewers using a uniform Excel template. The template captured the following headings: i) source of document including web address where relevant; ii) full citation of document; iii) scope of document – national or subnational; iv) focal sector(s) – housing, urban planning, infrastructure, agriculture, etc. v) findings on access to health resources; vi) findings on access to food and nutrition; vii) findings on access to other resources.

Social inclusion and equity were analysed through the lenses of access to health and nutrition resources in urban development. Narrative synthesis of data was performed along the main themes that emerged from the review of documents.

## Findings

With respect to access to health services 14 of the policy documents reviewed addressed issues related to health but only eight of these documents were explicit about access to health services. Table 1 highlights the key findings on health from the 14 documents.

**Table 1:** Consideration of access to health care within urban development policies in Nigeria

Policy/Strategy/Plan	Considerations of health and equitable access to health care
**Explicit policies and plans**	
1. Nigerian Economic Sustainability Plan (2020)	Includes measures to strengthen the response to COVID-19 & emergency preparedness; guarantee access to quality health service through primary health centres and expansion of social health insurance to all citizens; and boost research and development towards local production of medicines & pharmaceutical commodities
2. National Integrated Infrastructure Master Plan, 2015	One of the priority project portfolios that would receive urgent attention over the first 5 years of the plan is social infrastructure in which priority investments would be in construction of facilities including hospitals
3. National Urban Development Policy, 2012	The eighth (8th) out of 13 specific objectives of the policy will ensure provision of adequate, efficient and functional infrastructure and social services (including health services) in all human settlements
4. Nigeria Water Sector Road Map, 2011	Key strategic target in the area of sanitation which indirectly affects health
5. Nigeria Urban Reproductive Health Initiative, NURHI (2009–2020)	Seeks to eliminate supply- and demand-side barriers to access to contraceptives, and promote family planning into a social norm in Nigeria. Target is to achieve 36% contraceptive prevalence rate (CPR) by 2018.
6. Making Nigeria Open-Defecation-Free by 2025: A National Road Map	The plan outlines strategies for improving access to sanitation facilities particularly in densely populated urban settlements and squatter areas, by utilizing and implementing appropriate and sustainable technology
7. National Environmental Sanitation Policy (2005)	Promotion of public health and quality of life, ensuring adequate environmental sanitation and adequate portable water supply.
8. The National Water Sanitation Policy (2004)	It aims for improved access for all Nigerians to adequate, affordable, and sustainable sanitation. It recognizes the critical role of capacity building in promoting schools and community education in personal, water, and food hygiene.
**Implicit policies and plans**	
9. Agricultural Transformation Agenda Support Programme (2013–18)	The project rehabilitated 14 Community Health Centres, and provided 63 potable water and sanitation systems
10. The Nigeria Zero Hunger Strategic Plan (2017–2030)	Included in the approaches for achieving the SDG target 2 is the integration of direct nutrition interventions to the PHC under one roof initiative of the Federal Government (integrated service delivery)
11. Lagos Megacity Project (2005).	Part of the demands of the projects was the provision of health facilities, sanitation and water facilities
12. Lagos Metropolitan Development (2007)	
13. Inclusive Basic Service Delivery and Livelihood Empowerment Integrated programme, 2016	The programme aims to improve access for the poor and vulnerable to basic social and health services like water, sanitation, hygiene, within strengthened safety net systems
14. Urban Water Supply and Sanitation Improvement Project	The project aimed to improve access to clean water and sanitation for an estimated 1.5 million people in Taraba and Oyo States

On nutrition, 17 documents addressed issues on food and nutrition but 14 were explicit about acess to food/nutrition resources in urban areas. Table 2 highlights the key findings on nutrition from the 17 documents.

**Table 2:** Consideration of access to nutrition within urban development policies in Nigeria

Policy/Strategy/Plan	Considerations of equitable access to nutrition
**Explicit policies and plans**	
1. Nigerian Economic Sustainability Plan, 2020	Mass Agricultural Programme aims to improve local farming and agro-allied activities by adding up to 100,000 ha of new agricultural land to each State. Aim is to ensure food security through improved food production.
2. Special Agro-Industrial Processing Zones (SAPZ), 2020	SAPZ seeks to develop brownfield areas with critical infrastructure for sustainable agricultural outputs. The project will scale up food security in urban cities, and ensure strategic urbanization and economic security
3. National Integrated Infrastructure Master Plan, 2015	The plan prioritizes development of the agriculture sector and investments in staple crop processing zones, agro-industrial parks, and agricultural processing facilities, to contribute to food security
4. National Housing Policy, 2012	A policy strategy which will incorporate micro-enterprises (including agro-allied) ventures into the housing scheme could contribute to improving access to food and nutrition in urban settlements
5. Nigeria Water Sector Road Map, 2011	Key strategic target to be achieved in the medium term in the area of agriculture could indirectly address nutrition
6. The Nigeria Zero Hunger Strategic Plan (2017–2030)	Included in the approaches for achieving SDG 2 are: i) safety nets – cash transfers and school feeding – that provide access to nutritious foods to poor and vulnerable women and their families; and (ii) food/agricultural diversification
7. Nigeria Industrial Revolution Plan, 2014	Aims to maximize benefits from agricultural resources and boost local food production to meet local demands, through mid-stream and downstream procession and marketing activities.
8. National Environmental Sanitation Policy (2005)	Promotion of public health and quality of life, ensuring food sanitation for addressing food security.
9. Economic Recovery and Growth Plan (2017–2020)	Objectives of social inclusion will upscale home grown school feeding programme to provide a meal a day to at least 6 million primary school children. This will also strengthen the agricultural sector towards food security
10. Inclusive Basic Service Delivery and Livelihood Empowerment Integrated programme, 2016	The programme aims to improve access for the poor and vulnerable to food security, among others, within a strengthened safety net system
11. Agricultural Transformation Agenda Support Programme (2013–18)	The project sought to promote food and nutrition security by investing in the agricultural value chain and market linkages. It rehabilitated irrigation canals, crop production schemes, feeder roads & community markets
12. Agricultural Transformation Agenda, 2011	Accelerate achievement of food and nutritional security by re-structuring fertilizer procurement and distribution (towards private sector-led), marketing institutions, financial value chains and agricultural investment framework; and the focus will include youth and women.
13. Gender and Markets Initiative (2017)	Seeks to strengthen involvement of women in food vending for food security and economic empowerment
14. Livelihood Improvement family Enterprise (2016–19)	Promoted community-based on-farm and off-farm business activities along key agricultural value chain as a mechanism for job and wealth creation amongst unemployed youth and women
**Implicit policies and plans**	
15. National Social Protection Policy, 2016 (Draft)	Policy objective is to improve food security and nutrition towards inclusive growth and equality
16. Lagos Megacity Project (2005).	Part of the demands of the projects was the provision of decent markets through infrastructural upgrade.
17. Lagos Metropolitan Development (2007)	

The findings on equity and social inclusiveness of the urban development policies/plans are subsequently structured into two main sub-sections, access to health resources and access to food and nutrition resources. In each sub-section, we start by highlighting the thematic areas that reflect potential policy contributions to social inclusion and equitable access to health or nutrition. Then, in chronological order, we present the findings from each document in the areas of health or nutrition.

### Equity and social inclusion in access to health resources within urban development policies and plans

Focusing on the eight urban development policies/plans that were explicit on health and public health issues, five thematic areas that represent potential contributions to equity and social inclusion in access to health resources emerged, and these were: (i) Provision of functional and improved health infrastructure; (ii) Primary Health Care (PHC) system strengthening for quality health services; (iii) Provision of safety nets and social health insurance; (iv) Community participation and integration; and (v) Public education and enlightenment.

#### Provision of functional and improved health infrastructure

Provision of health and public health infrastructure appeared to be a recurrent theme among urban development policies and plans in Nigeria. This was reflected in five policies/plans as assessment of exisiting facilities and construction of more facilities or renovation of existing ones across the country.

In the* National Integrated Infrstructure Master Plan (NIIMP, 2015).* infrastructural plans for the Nigerian health sector include the establishment of a minimal number of functional primary healthcare clinics linked to a contiguous general hospital in each LGA, and a functional general hospitals in every LGA manned by qualified personnel, with strong referral system to contiguous tertiary hospitals. In addition, existing tertiary and specialist hospitals will be revamped to meet local population needs, and quaternary mono-specialist centers will be distributed to ensure equitable access to all sections of the country [15]. These will contribute to an integrated health system with infrastructure that guarantees high quality, affordable and sustainable world-class healthcare services for all.

The *National Urban Development Policy (NUDP, 2012)* states that in the context of social welfare services, access to health services for urban dwellers will be achieved by ensuring adequate provision of improved health facilities to all categories of urban residents, particularly, poor and vulnerable groups such as women, children, the aged and the disabled [16]. Some of the strategies outlined for achieving equitable access to health services include assessment of existing facilities, provision of additional ones to fill any gaps, and establishment of effective, efficient, and functional healthcare delivery that is easy to access to citizens.

The *National Road Map for Making Nigeria Open-Defecation Free by 2025* provides the pathway for achieving an open defecation free Nigeria by providing sanitation facilities in public spaces (that are typically found in urban areas). It proposes the siting of a re-structured version of Sanicentres (Sanitation Resource Centre/Sanitary Mart/Sanitary Hub) at local government and ward levels [17].

The *Water Sector Road Map (2011)* has that one of its key strategic targets to be achieved in the medium term is to increase access to sanitation facilities from 32 to 65% in the country [18]. While the tasks states and local governments to improve access to sanitary facilities by contructing public toilets [19].

#### PHC strengthening for quality service delivery

A few policies/plans highlighted strategies that will contribute to strengthening the primary health care (PHC) system to deliver quality health services through capapcity strengthening and harmonization of human resources for health, improved data and information systems, sustainable supply of medicines and medical products through local production, and public-private partnerships.

The *Nigeria Economic Sustainability Plan (NESP, 2020)* seeks to improve emergency preparedness and response in the country by equipping primary health centres to guarantee access to quality health services in urban areas that were hardest hit by the COVID-19 pandemic, and rural areas as well [20]. It also talks about boosting research and development towards local production of medicines and pharmaceutical commodities in Nigeria.

The specific aspirations and targets of the health objective of the social infrastructure master plan of *NIIMP (2015)* include to revitalize public healthcare services by improving human resource capacity (number and skills) at the PHC level and harmonization to ensure efficient utilization of limited HRH availability at that level [15]. The plan also aspires to provide sustainable influx of inputs from local production of medicines and vaccines to address the issues of frequent stock-outs in primary health centres. It will also strengthen health management information system to generate timely data for health decision making and service improvement. Furthermore, it aspires to increase public-private partnership in provision of sustainable primary health care services.

The *Nigeria Urban Reproductive Health Initiative (NUHRI, 2009-2020)* seeks to eliminate barriers to accessing contraceptives by making commodities available at all times in primary health centers [21].

#### Provision of safety nets and social health insurance

In a bid to address the health and economic challenges facing Nigeria as a result of the global COVID-19 pandemic the *NESP (2020)* highlights that access to social health insurance will be expanded to all citizens including vulnerable groups, women, and those with disabilities [20].

#### Community participation and integration

Community participation and social integration in urban development appeared to also cut across several of the policies and plans.

The *NUDP (2012)* recognizes social integration in its health-related thrust, and the policy clearly states that its implementation will promote social participation and integration in urban centres to achieve an inclusive city and foster national unity [16].

The *National Road Map for Making Nigeria Open-Defecation Free by 2025* highlights community-led total sanitation (CLTS) as one of the approaches for achieving an open defecation free Nigeria [17]. In this instance, community demand and preference (including capacity to maintain) will be taken into consideration in the design of Sanicenters.

Similarly, the *National Water Sanitation Policy (NWSP, 2004)* which aims for improved access for all Nigerians to adequate, affordable, and sustainable sanitation, will be achieved through the active participation of communities, households, and individuals. The policy recognizes community ownership and participatory approach, and identifies the need to involve communities in choosing sanitation measures that are culturally acceptable as well as affordable to them. Furthermore, households and individuals are responsible for their compliance with sanitary regulations and requirements [22].

Through increased and better community engagement in family planning services, the *NURHI (2009-2020)* supports communities to confront and address the cultural and social barriers to the use of modern methods of contraception [21].

#### Public education and enlightenment

The *National Road Map for Making Nigeria Open-Defecation Free by 2025* provides the pathway for achieving an open defecation free Nigeria using different approaches including capacity development of sanitary workers, and media campaigns to influence behaviour change in communities [17].

The *National Environment Sanitation Policy (2005)* pushes for the training and re-training of environmental health professionals as well as public education on sanitation measures through zonal seminars, and community for a [19].

The *NSWP (2004)* recognizes the critical role of capacity building in promoting schools and community education in personal, water, and food hygiene. Hence, its implementation calls for training of teachers, pupils and community health workers to promote hygienic practices in schools and communities [22].

### Equity and social inclusion in access to food and nutrition resources within urban development policies and plans

Focusing on the 14 urban development policies/plans that were explicit on food and nutrition, five (5) themes that represent potential policy contributions to equity and social inclusion in access to food and nutrition resources also emerged, and these were: (i) Provision of accessible and affordable land to farmers; (ii) Upscaling local food production, diversification and processing (downstream and mid-stream); (iv) Private-sector participation; (v) Provision of safety nets; and (v) Special considerations for vulnerable groups.

#### Provision of accessible and affordable land to farmers

Five (5) policies/plans underline strategies to promote access to food and nutrition through increased land ownership by local farmers. This will be achieved by increasing the land that is available for agriculture and making it more affordable to farmers.

The *NESP (2020)* specifically provides for the implementation of a Mass Agricultural programme that aims to improve local farming and agro-allied activities by adding 20,000–100,000 ha of new agricultural land to each State of the federation [20].

The *Special Agro-Industrial Processing Zones (2020)* project seeks scale up food security in urban cities, and ensure strategic urbanization and economic security by developing brownfield areas with critical infrastructure for sustainable agricultural production, and making these areas accessible to farmers [23].

The *NIIMP (2015)* prioritizes development of the agriculture sector and investments in staple crop processing zones, agro-industrial parks, and agricultural processing facilities. One of its aspirations is to secure sustainable food security for all Nigerians through an increased percentage of arable land [15].

The *National Housing Policy (2012)* plans to incorporate agro-allied ventures in the housing scheme by making land ownership available and accessible at an affordable price to individuals (including farmers) for small-scale and subsistent farming. This will contribute to improving food security for low-income urban dwellers [24].

The *Water Sector Road Map (2011)* medium-term strategic target was to increase total irrigable land by 50% by 2015 across the country [18]. This may have contributed to improving access to food and nutrition, however, there is no evidence from the project evaluation to validate this potential impact.

#### Up scaling local food production, diversification and processing

Agricultural diversification and improved local food production and processing resonated through three (3) policies/plans. Facilities (including equipment, stems/seedlings and guidelines) to enable local production and processing of foods will be made available and affordable to local farmers.

The *Nigeria Zero Hunger Strategic Plan (2017-2030)* provides a road map for tracking progress in implementation of priority actions for achieiving zero hunger by 2030 in Nigeria. Specific approaches that address access to food and nutrition include food/agricultural diversification focusing on provision of enhanced cassava, orange-fleshed sweet potato, and soybean stems/seedlings [25].

The *Nigeria Industrial Revolution Plan (2014)* is Nigeria’s first comprehensive initiative that focuses on improving downstream and mid-stream processing of industrial resources of the country [26]. It seeks to harness the country’s rich agricultural ecosystem and revitalize availability of food and nutrition by providing facilities for increased local production and processing of sugar, palim oil and cocoa.

The *National Environmental Sanitation Policy (2005)* had as its objective to develop guidelines on sound food sanitation practices to ensure that locally produced and processed food is safe and wholesome for consumption [19]. These guidelines will stipulate operational standards for food handlers, capacity requirements of environmental health officers, and regulations for monitoring food sanitation practices.

#### Provision of safety nets

Provision of safety nets in the form of cash transfers and food packages to vulnerable groups, specifically children and victims of insurgency, also stood out in some of the plans.

The *Economic Recovery and Growth Plan (2017-2020)* seeks to strengthen the agricultural sector towards food security through a socially inclusive strategy of upscaling home-grown school feeding programme to provide a meal a day to at least 6 million primary school children [27].

The *Nigeria Zero Hunger Strategic Plan (2017-2030)* also seeks to address access to food and nutrition resources through the provision of safety nets in form of cash transfers and school feeding to improve access to nutritious foods to poor and vulnerable women and their families [25].

The *Inclusive Basic Service Delivery and Livelihood Empowerment Integrated programme (2016)* provides safety net systems in form of cash transfers and food packages to individuals and households in north-east Nigeria [28]. This is to cushion the effects of the Boko Haram insurgency on access to food and nutrition resources.

#### Private-sector participation

Private-sector participation was only prominent in the *Agricultural Transformation Agenda (ATA, 2011)* and the focus is on creating eco-systems where small, medium, and large scale farmers co-exist and flourish. The Agenda seeks to accelerate achievement of food and nutritional security by re-structuring fertilizer procurement and distribution towards private sector-led procurement and distribution to eliminate limitations in access arising from frequent stock-outs and government bureaucracies [29].

#### Special considerations for vulnerable groups

There were some special considerations for vulnerable groups in some plans/programmes that addressed issues of access to food/nutrition resources.

The empowerment of young people and women to have access to agricultural products is underlined in the *ATA (2011)* as a strategy for accelerating achievement of food and nutritional security in the country [29].

Similarly, the *Livelihood Improvement Family Enterprise (2016-19)* sought to promote community-based on-farm and off-farm business activities along key agricultural value chain as a mechanism for job and wealth creation for unemployed youth and women [30].

Two (2) other plans specifically seek to cushion the effects of the Boko Haram insurgency on poor and vulnerable groups in north-east Nigeria. The *Inclusive Basic Service Delivery and Livelihood Empowerment Integrated programme (2016)* outlines specific strategies that aim to improve access to food security for poor and vulnerable groups, and to reduce poverty and vulnerability in the region [28].

The *Gender and Markets Initiative* was birthed in 2017 due to the increase of urban population in Maiduguri as a result of Borno insurgence. Their major focuses is on gender, food and nutrition so as to Increase and strengthen the involvement of women in food vending as a source of food security and economic empowerment.

## Discussion

Although Nigeria has elaborated various policies, strategies, and plans that could contribute to improving access to health and nutrition resources for urban dwellers, our review highlighted limitations in potential policy contributions to social inclusion and equitable access to urban resources. In a few of the policies that were reviewed, the considerations of health and nutrition are clearly defined (explicit), while in the majority they were either implicit or absent. There was less consideration of access to health services compared to access to food/nutrition in the policies and plans that were reviewed. Achieving social inclusion in urban development through efficient policies, strategies and plans is central to the SDGs. Unfortunately, Nigeria is ranked 160 of 166 on the SDGs dashboard, with a score of 49.28 [31]. The implication of these is that Nigeria is lagging behind many countries in making progress towards achievement of SDGs. Ensuring that every citizen of the country has equal access to health and nutrition is important for achieving the Sustainable Development Goals, especially Universal Health Coverage (UHC), which aims to ensure healthy lives, promote well-being for all at all ages by 2030.

Although issues on health are located within urban development policies in Nigeria, policy contributions to social inclusion and equitable access to health services are not prominent. Less than one-third of the policy documents and plans elaborated strategies for ensuring inclusivity and equity, and only one document highlighted safety net and social health insurance as a strategy. Furthermore, considerations on health were more focused on the construction of more health facilities without clear statements on how these facilities will be located in ways that ensure equitable access. Evidence shows that utilisation of health services is constrained by poor siting of health facilities [32]. Moreover, when facilities are located in places far from service users, it raises the cost of access, delays the time to access services, and discourages users [33]. This contributes to the wide health equity gap within residents in urban areas, and this can be far worse for women, poor, and underserved groups.

On access to food and nutrition resources, the policies were more detailed about measures to be taken to ensure increased food production and better access to food and nutrition for vulnerable groups such as women and children. Some of the policies also highlighted the linkage between the health and agriculture sectors by making provisions for integrating nutrition interventions into primary health care settings. Although some policies and plans highlighted measures to improve access to food and nutrition, it is noteworthy that the country’s key policy for sustainable urban development – that is the National Urban Development Policy, 2012 – does not consider issues of access to food and nutrition in urban planning and development. In the current efforts to revise and update the policy, access to food and nutrition, as well as other public goods, should be considered.

Gaps still exist between access of the poor and non-poor on urban services and resources in Nigeria [34]. Although there is limited evidence to assess the effectiveness of these policies and plans in improving access to health services, and food and nutrition to urban dwellers, there is the general assertion that many of these policies have failed to achieve their stated goals and objectives. Urban centres are apparently the worst hit by poverty owing to less opportunities to engage subsistence farming, competing demands on an already battered finance by poverty levels, and fierce competition for available resources which only the “fittest” emerges winner. Successive governments in Nigeria have attempted solving the problem of exclusion in healthcare and food/nutrition through inclusive education and provision of equal learning opportunities for able and disabled children, based on the premise that education enhances capacity to contribute to economic development in the areas of health, food, and agriculture [35]. However, the inequities have not be adequately addressed, and are widening in urban areas.

The potential influence of political interference in urban development must be underscored. The frequent adjustments in ministerial portfolios, by removing, adding, or merging some ministries, contribute to complicating roles and responsibilities of individuals, groups and organizations in urban development in Nigeria. For instance, the merging of agencies could result in the cessation of existence of some functions which are critical. Similarly, the lack of operational frameworks/guidelines for implementation of urban development policies in Nigeria has been underscored. Designing or adapting exising framworks may guide policy makers in the recognition of the needs of urban dwellers and in the selection of appropriate strategies for addressing these needs and achieving social inclusion. City Resilience Framework (CRF) was used in the development of the Lagos Resilience Strategy, LRS 2020. CRF includes the four dimensions of leadership and strategy, health and wellbeing, infrastructure and environment, and economy and society. The LRS then blends the CRF with the SDGs in driving the strategy for developing Lagos and could serve as a guide in developing Nigerian urban policies.

Social inclusion in urban development has not been extensively researched. Other studies have focused on specific groups such as ethnic and religious minorities rather than how social inclusion is mainstreamed within public sector policies. This desk review is one of the first in Nigeria to critically assess urban development policies and plans with a social inclusion lens. It highlights important policy gaps that need to be addressed in order to pave way for equitable access to urban resources for urban dwellers. However, the assessment is limited in its reliance on secondary data obtained from documents alone. The findings would have been enriched through primary data collection from those who formulated the policies and strategies, as well as the policy implementers and potential beneficiaries. Furthermore, some of the documents in the review had applications to all geographic settings, beyond the scope of sustainable urban development.

## Conclusions

The findings from this review underscore the need for a revision of Nigeria’s urban development policies and the development of implementation guidelines that align to the SDG targets for ensuring social inclusiveness in access to urban resources. There is minimal consideration of access to health services compared to access to food and nutrition in urban development policies, plans and strategies in Nigeria. Some policies, plans and strategies were explicit on issues of access to health and nutrition resources in urban areas while others were implied. Where access to health services was considered, equitable access was not apparent. On the other hand, policies that addressed food and nutrition issues focused on measures to adopt to increase food production and ensure access to food and nutrition for vulnerable groups including women and children; although most of these policies were not urban-centric. As the urban population continues to grow in Nigeria, national and subnational governments have an increasing task of ensuring equitable access to urban resources, particularly healthcare and food/nutrition.

## Supplementary Information

**Additional file 1.** Overview of geographic scope, goals and sectors addressed in the urban development policies/strategies/plans included in the review.

## Data Availability

All data generated and analysed during this study are included in this published article and its supplementary information file.

## Abbreviations

ATA: Agricultural Transformation Agenda
CRF: City Resilience Framework
LRS: Lagos Resilience Strategy
NESP 2005: National Environment Sanitation Policy (2005)
NESP 2020: Nigeria Economic Sustainability Plan (2020)
NIIMP: National Integrated Infrastructure Master Plan
NUDP: National Urban Development Policy
NURHI: Nigeria Urban Reproductive Health Initiative
NWSP: National Water Sanitation Policy
