# Supplementary material for: Analysis of equity and social inclusiveness of national urban development policies and strategies through the lenses of health and nutrition
Source: Int J Equity Health. 2021 Apr 16;20:101. doi: 10.1186/s12939-021-01439-w (PMC8051828; doi:10.1186/s12939-021-01439-w)
Supplement: Supplementary file 1 — Additional file 1. Overview of geographic scope, goals and sectors addressed in the urban development policies/strategies/plans included in the review. [file 12939_2021_1439_MOESM1_ESM.docx]

**Supplemental file: Overview of geographic scope, goals and sectors addressed in the urban development policies/strategies/plans included in the review**

| **Policy/Strategy/Plan** | **Geographic scope** | **Policy Goals/Aims/Vision/Motivation** | **Sectors addressed** |
| --- | --- | --- | --- |
| 1. National Urban Development Policy, 2012 | National | Promote a dynamic system of clearly defined, **planned and well-managed urban settlements**, which foster **sustainable economic growth**, promote efficient and balanced urban and regional development, and ensure **improved standard of healthy living** for all Nigerians. | Housing, Infrastructure, Economy |
| 1. National Housing Policy, 2012 | National | **Revitalization of the housing sector** to serve as a catalytic instrument for ensuring **rapid and effective socio-economic development.** | Housing, Economy |
| 1. National Urban and Regional Planning Decree, 1992 | National | Facilitate the preparation and implementation of development plans and planning schemes to create **better environment for living, working and recreation** | Housing, Infrastructure |
| 1. Nigeria Industrial Revolution Plan, 2014 | National | Drive a process **of intense industrialization**, based on sectors where Nigeria has comparative advantage | Agriculture; Solid minerals; Oil & gas; Etc |
| 1. Nigeria Water Sector Road Map, 2011 | National | Articulate FGN’s objective of **developing the nation’s water resources** towards actualization of the sector’s potentials in the short, medium and long term | Water |
| 1. Agricultural Transformation Agenda, 2011 | National | Achieve a **hunger-free Nigeria** through an agricultural sector that drives **income growth**, accelerates achievement of **food and nutritional security**, generates **employment** and transforms Nigeria into a **leading player in global food markets** | Agriculture, Food and nutrition, Economy, Employment |
| 1. Agricultural Transformation Agenda Support Programme (2013-18) | National | Promote employment generation and shared wealth creation along the commodity value chains, as well as food and nutrition security. | Agriculture, Food and nutrition |
| 1. Making Nigeria Open-Defecation-Free by 2025: A National Road Map | National | Reverse the country’s losses due to prevalence of open defecation. Ensure the country makes progress towards achieving the milestone on sanitation coverage set by the Draft National Water-Sanitation Policy, 2004; Nigeria’s commitment to SDG 6 and PEWASH strategy. | Sanitation, Housing, Infrastructure |
| 1. National Integrated Infrastructure Master Plan, 2015 | National | Blue-print for boosting and modernizing the nation's stock of Infrastructure, over the next 30 years (2045). Anchored on the need to harmonize sectoral infrastructure plans considering inter-sectoral linkages. Framework to guide interventions, investments, and budgetary allocations to the sector till 2045. | Infrastructure (all sectors) |
| 1. Nigeria Economic Sustainability Plan, 2020 | National | Address the health and economic challenges facing Nigeria as a result of the global COVID-19 pandemic, & foster new ways of working, producing, learning, and managing public health and safety in the coming years | All sectors – Education, Agriculture, Housing, Health, WASH, Energy, Employment |
| 1. Economic Recovery and Growth Plan (2017-2020) | National | Restore economic growth by focusing on macroeconomic stability and economic diversification. Foster social inclusion and economic growth through job creation and human capital investment. Build a globally competitive economy through improvements in infrastructure and business environment. | Employment (human capital investment), Economy, Infrastructure |
| 1. The Nigeria Zero Hunger Strategic Plan (2017-2030) | National | Articulates what Nigeria must do to achieve SDG 2 (zero hunger) by 2030. A road map for tracking progress in implementation of priority actions such as safety nets, agricultural diversification, and integration of direct nutrition interventions into the Primary Health Care Under One Roof (PHCUOR) initiative | Agriculture (food and nutrition), Health |
| 1. Nigeria Urban Reproductive Health Initiative, NURHI (2009-2020) | State – Oyo, Kwara, Kaduna, Edo, Lagos | Eliminate supply- and demand-side barriers to use of contraceptives. Promote family planning to become a social norm in Nigeria. Align with the country’s goal of 36% Contraceptive Prevalence Rate (CPR) by 2018. | Health |
| 1. National Social Protection Policy, 2016 (Draft) | National | Motivation – Realization that economic growth does not equate to social protection. Goal: Promote inclusive growth, equality and social security to ensure a life of dignity for all Nigerians | Employment, Economy, Agriculture Infrastructure, Social services |
| 1. Special Agro-Industrial Processing Zones (SAPZ), 2020 | National scope – Regional implementation | Develop **brownfield areas with critical infrastructure for sustainable agricultural production and better livelihood of residents. Improve access to agro products and promote economic independence. | Agriculture, Infrastructure |
| 1. Lagos Megacity Project (2005). 2. Lagos Metropolitan Development (2007) | State level –Lagos | Transform Lagos State into a world-class city through the provision of critical infrastructure including affordable housing, health facilities, sanitation and water facilities; with a particular focus to nine (9) slum areas. | Housing, Water, Public health (sanitation) |
| 1. Gender and Markets Initiative (2017) | Metropolitan – Maiduguri city | Increase and strengthen the involvement of women in food vending as a source of food security and economic empowerment | Gender, Food and Nutrition |
| 1. Livelihood Improvement family Enterprise (2016-2019) | National – 774 LGAs | Promote community-based on-farm and off-farm businesses along key agricultural value chain for wealth creation among unemployed youth & women | Economy, Employment, Agriculture |
| 1. Inclusive Basic Service Delivery and Livelihood Empowerment Integrated programme, 2016 | Regional – North East | Improve access for the poor and vulnerable to basic social and health services like water, sanitation, hygiene, education, livelihood opportunities, food security, to curb the effects of the insurgency. Strengthen safety net systems | Health & Public health Education, Economy, Employment, Food security |
| 1. Urban Water Supply and Sanitation Improvement Project | State level – Oyo and Taraba | Improve access to clean water and sanitation for an estimated 1.5 million people in Taraba and Oyo States | Water |
| 1. National Environmental Sanitation Policy (2005) | National | It has among its goals the promotion of public health and quality of life, ensuring adequate environmental sanitation, adequate solid waste management, excreta, and sewage management, market and abattoir sanitation, pest and vector control, school sanitization and adequate portable water supply and proper housing . | Sanitation, Public Health |
